# Cardiac arrest due to acquired long QT syndrome during gynecologic laparoscopy: a case report

**DOI:** 10.3389/fmed.2026.1857566

**Published:** 2026-06-03

**Authors:** Wenwen Wang, Ruijing Wang, Guangwei Li, Yun Zhang

**Affiliations:** 1 Department of Anesthesiology, The 960th Hospital of PLA, Jinan, Shandong, China; 2 Department of Vascular Surgery, The 960th Hospital of PLA, Jinan, China; 3 Department of Anesthesiology, Jinan Municipal Hospital of Traditional Chinese Medicine, Jinan, Shandong, China

**Keywords:** cardiac arrest, dexmedetomidine, female, hypokalemia, long QT syndrome, perioperative period

## Abstract

**Background:**

Acquired long QT syndrome (aLQTS) is a disorder of delayed myocardial repolarization induced by medications, electrolyte disturbances, and other factors, with a significantly higher risk in females than in males. Various perioperative factors can trigger aLQTS, which may lead to cardiac arrest in severe cases, yet clinical recognition remains challenging.

**Case summary:**

This article reports a 38-year-old female patient who underwent laparoscopic combined hysteroscopic tubal lavage under general anesthesia for “bilateral tubal obstruction.” During the procedure, the patient suddenly developed a heart rate of 40 beats per minute, followed by torsade de pointes (TdP) that rapidly progressed to cardiac arrest. The patient was successfully resuscitated after timely cardiopulmonary resuscitation, defibrillation, and pharmacological interventions. Postoperative electrocardiogram and 24-h Holter monitoring showed progressive prolongation of the QTc interval, reaching a maximum of 581 ms. Follow-up electrocardiogram at 1 month post-surgery showed that the QTc interval had returned to normal (421 ms). Based on a review of the literature, the final diagnosis was aLQTS.

**Conclusion:**

aLQTS is one of the important causes of perioperative cardiac arrest. Female sex, electrolyte disturbances, bradycardia, and QT-prolonging medications can act synergistically as triggers. Comprehensive interventions including early recognition of abnormal electrocardiographic signals, timely resuscitation, and correction of precipitating factors are key to improving prognosis.

## 1 Introduction

Long QT syndrome (LQTS) is a group of cardiac ion channel disorders characterized by prolonged QT interval on electrocardiogram, T-wave abnormalities, and susceptibility to torsade de pointes (TdP), with a prevalence of approximately 1:2000 (1). Acquired LQTS (aLQTS) is more common than congenital LQTS (2). Multiple perioperative medications and physiological changes can serve as triggers for aLQTS, which can lead to cardiac arrest in severe cases. This article reports a case of a middle-aged woman who developed cardiac arrest due to aLQTS during gynecological laparoscopic surgery and was successfully resuscitated, and to discuss its prevention and treatment strategies in light of the latest literature, as well as to emphasize the importance of recognition and management of aLQTS by perioperative anesthesiologists and nursing staff.

## 2 Case report

### 2.1 General information

The patient, a 38-year-old female, 167 cm in height, weighing 80 kg, was admitted with “secondary infertility and bilateral tubal obstruction.” Chief complaints: failure to conceive after 4 years of unprotected intercourse, decreased menstrual volume for 6 months. Past medical history: underwent hysteroscopic surgery for “endometrial polyps” in 2009 with good postoperative recovery. Denied history of hypertension, diabetes mellitus, heart disease, syncope, or seizure-like episodes. No family history of cardiac disease or sudden death.

### 2.2 Admission physical examination and laboratory tests

Vital signs: temperature 36.2 °C, pulse 76 beats/min, respiration 19 breaths/min, blood pressure 114/78 mmHg.

Complete blood count: hemoglobin 124 g/L, platelets 324 × 10^9^/L, white blood cell count normal, all other indicators are within the normal range.

Biochemistry: sodium 138 mmol/L, calcium 2.31 mmol/L (normal range 2.11 ~ 2.52 mmol/L), potassium 3.9 mmol/L, urea nitrogen 6.42 mmol/L, creatinine 52.6 μmol/L, all other laboratory parameters were within normal ranges.

Coagulation function and thyroid function were normal.

**Figure 1:**
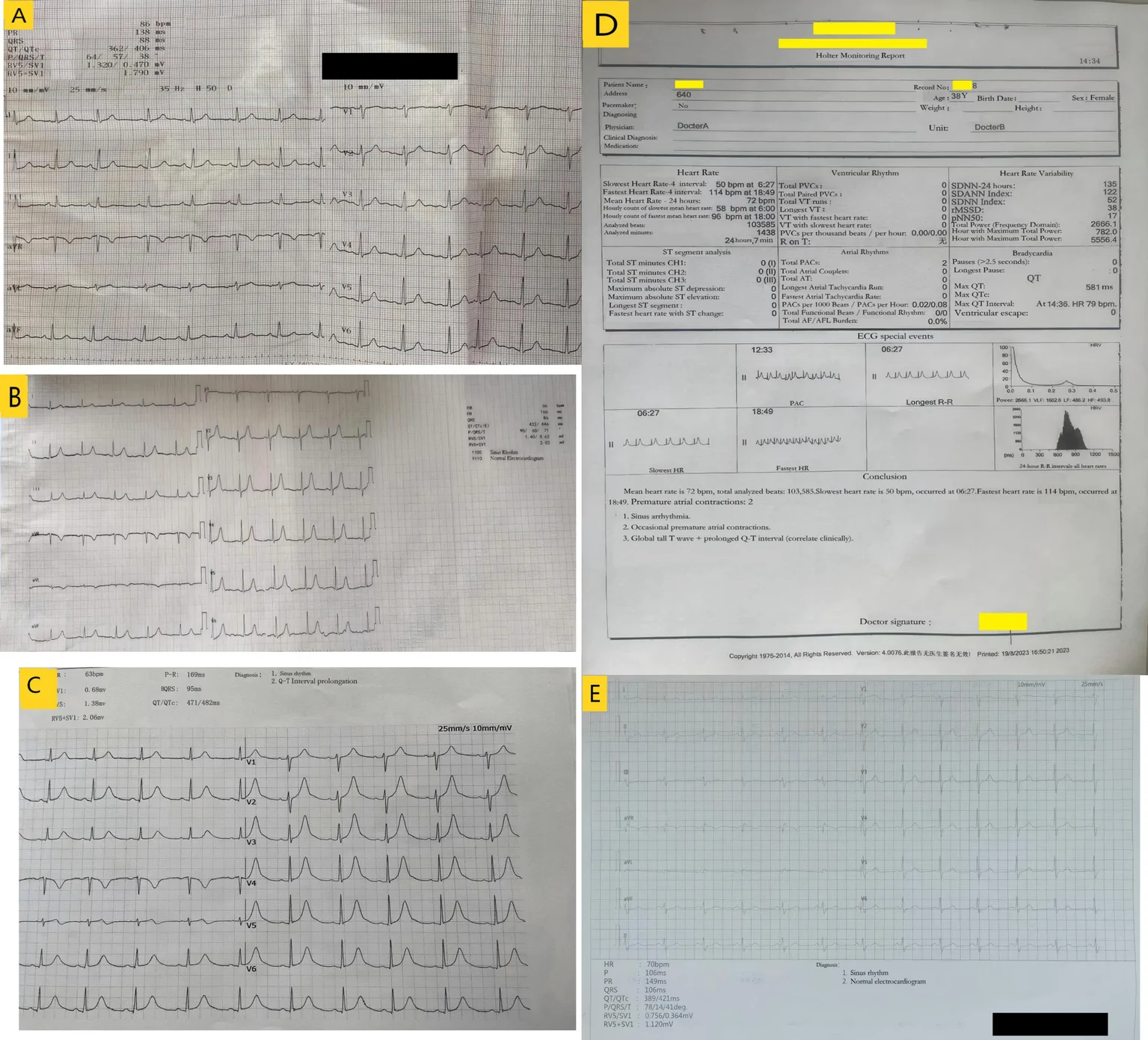
Perioperative electrocardiographic findings. **(A)** Preoperative ECG showing normal QTc interval (406 ms). **(B)** ECG at 4.5 h post-resuscitation showing tall T waves and QTc interval (446 ms). **(C)** ECG at 34 h post-resuscitation showing QTc interval (482 ms). **(D)** 24-h Holter recording on day 3 revealing maximum QT interval of 581 ms. **(E)** Follow-up ECG at 1 month showing normalized QTc interval (421 ms).

Electrocardiogram: sinus arrhythmia, heart rate 76 beats/min, QT/QTc 362/406 ms (All QTc interval values in this case were calculated using the Bazett formula, Figure 1A).

### 2.3 Surgical and anesthesia process

On the day of surgery at 9:22, laparoscopic combined with hysteroscopic tubal hydrotubation was performed under general anesthesia. Anesthesia induction: dexmedetomidine loading dose 80 μg (infused over 10 min), midazolam 5 mg, propofol 100 mg, vecuronium 8 mg, sufentanil 15 μg. Induction was smooth, tracheal intubation was successful, and sevoflurane 2% was administered for maintenance, with dexmedetomidine continuously infused at 0.5 μg/(kg·h). After induction, blood pressure was 96/65 mmHg, and fluid infusion was appropriately accelerated.

At 9:58, surgery began; vital signs were stable, blood pressure 110–117/80–85 mmHg, heart rate 52–57 beats/min.

### 2.4 Cardiac arrest and resuscitation process

At 10:20, the patient’s heart rate suddenly dropped to 40 beats/min. Dexmedetomidine infusion was immediately discontinued, and atropine 0.5 mg was administered with no effect, followed by torsade de pointes which rapidly progressed to cardiac arrest. Surgery was immediately halted, cardiopulmonary resuscitation was initiated, and epinephrine 1 mg and dexamethasone 10 mg were administered intravenously with continuous chest compressions.

At 10:25, the patient regained sinus rhythm, but with ventricular bigeminy and ST-segment depression; lidocaine 100 mg was administered intravenously. No defibrillation was delivered during the entire resuscitation process.

At 10:30, frequent premature ventricular contractions occurred, and lidocaine 50 mg was added.

At 10:33, premature ventricular contractions disappeared, but ST-segment depression persisted; blood pressure was 79/45 mmHg, and norepinephrine 8 μg was administered intravenously, followed by continuous infusion at 2 μg/(kg·min).

At 10:38, ST-segment normalized.

At 10:50, the patient regained consciousness with slightly weak limb strength.

### 2.5 Post-resuscitation laboratory tests

At 10:51, arterial blood gas analysis: pH 7.389, PCO₂ 30.2 mmHg, PO_2_ 408 mmHg, potassium 3.4 mmol/L (↓), sodium 133 mmol/L, calcium 0.98 mmol/L (↓↓), glucose 9.3 mmol/L, lactate 2.5 mmol/L, HCO_3_^−^ 17.8 mmol/L, BE −7.8 mmol/L, No abnormalities were found in the remaining indicators. Sodium bicarbonate 50 mL was administered as a rapid intravenous infusion.

At 11:00, muscle strength recovered, vital signs stable, endotracheal tube removed.

At 12:00, transferred to ICU.

At 13:03, repeat blood gas: potassium 3.9 mmol/L, calcium 0.99 mmol/L, lactate 1.3 mmol/L, BE −2.9 mmol/L.

At 14:39, electrocardiogram: sinus rhythm, tall T waves, QT/QTc 432/446 ms (Figure 1B).

At 16:37, cardiac enzyme panel: myoglobin < 2 ng/mL, creatine kinase 65 U/L, cardiac troponin 0.14 ng/mL, CK-MB (chemiluminescence) 4.8 ng/mL, CK-MB (mass assay) 1.7 U/L, all within normal range.

At 18:46, echocardiography: left ventricular end-diastolic diameter 4.57 cm, mildly reduced left ventricular systolic function, LVEF 50%, mild mitral regurgitation.

Other tests: white blood cells 17.6 × 10^9^/L (↑), D-dimer 2.35 mg/L (↑), antithrombin III 67.9% (mildly ↓), fibrin degradation products 6 μg/mL (↑); renal function, C-reactive protein, NT-proBNP were normal and other laboratory findings were unremarkable.

### 2.6 Postoperative electrocardiographic evolution

Day 1 post-surgery at 14:34: 24-h Holter monitoring initiated.

Day 2 post-surgery at 20:00: The patient complained of pain in the left scapular area. Repeat electrocardiogram showed sinus rhythm, tall T waves, QT/QTc 471/482 ms (Figure 1C). Repeat cardiac enzyme panel: myoglobin < 7.58 ng/mL, creatine kinase 47 U/L, cardiac troponin < 0.1 ng/mL, CK-MB (chemiluminescence) 2.2 ng/mL, CK-MB (mass assay) 1 U/L, all within normal range.

Day 3 post-surgery: 24-h Holter monitoring results showed sinus arrhythmia, occasional atrial premature beats (2 in 24 h), persistent tall T waves, and a maximum QTc interval of 581 ms (at a heart rate of 79 beats/min) (Figure 1D). The lowest heart rate was 50 beats/min (06:27), the highest heart rate was 114 beats/min (18:49), and the average heart rate was 72 beats/min.

The patient remained asymptomatic and was discharged on postoperative day 4.

### 2.7 Follow-up results

Postoperative 1-month outpatient follow-up: Repeat electrocardiogram showed sinus rhythm, heart rate 72 beats/min, QT/QTc 389/421 ms (Figure 1E), and electrolytes were normal. The patient stated: “I have no memory of the entire surgical procedure. After discharge, I felt that my body had largely recovered and my daily life was not affected. The doctor recommended genetic testing, but I declined”.

### 2.8 Clinical timeline

See Table 1.

**Table 1:** Clinical timeline of the case.

Time point	Event
Preoperative	A 38-year-old female (167 cm, 80 kg) admitted for secondary infertility. Preoperative ECG: sinus arrhythmia, QTc interval 406 ms.
Day of surgery 09:22	General anesthesia induction with dexmedetomidine (80 μg), midazolam, propofol, vecuronium, sufentanil.
09:58	Surgery started. Heart rate 52–57 bpm, BP stable.
10:20	Sudden heart rate drop to 40 bpm, followed by torsade de pointes and cardiac arrest.
10:20–10:25	CPR started; epinephrine 1 mg, dexamethasone 10 mg given.
10:25	Sinus rhythm restored with ventricular bigeminy and ST depression. Lidocaine 100 mg IV.
10:30	Frequent PVCs, additional lidocaine 50 mg.
10:33	PVCs resolved, ST-segment depression persisted, blood pressure was 79/45 mmHg, Norepinephrine given.
10:38	ST segment normalized.
10:50	Patient regained consciousness.
10:51	ABG: K^+^ 3.4 mmol/L, Ca^2+^ 0.98 mmol/L, BE −7.8 mmol/L.
11:00	Tracheal extubation.
12:00	Transferred to ICU.
Post-resuscitation 14:39	ECG: QTc interval 446 ms.
Postoperative day 2 20:00	QTc interval 482 ms.
Postoperative day 3	24-h Holter: maximum QTc interval 581 ms.
Postoperative day 4	Patient discharged.
1 month follow-up	ECG: QTc interval 421 ms, normal.

## 3 Discussion

### 3.1 Diagnosis of aLQTS and perioperative precipitating factors

**Figure 2:**
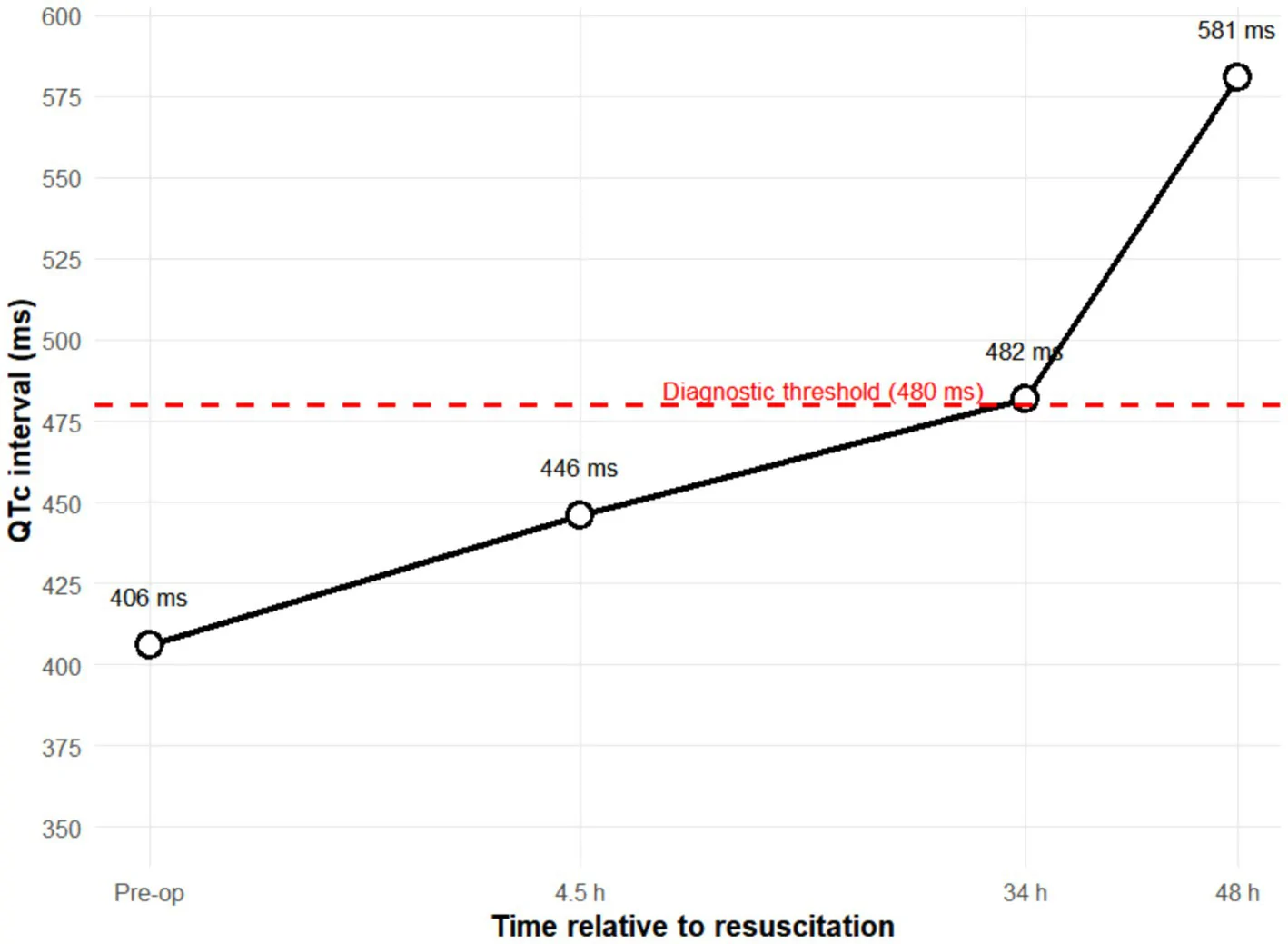
Dynamic changes in QTc interval during the perioperative period. QTc interval values are shown at four time points: preoperative baseline (406 ms), 4.5 h (446 ms), 34 h (482 ms), and 48 h (581 ms) after resuscitation. The red dashed line indicates the diagnostic threshold for LQTS in females (480 ms). The progressive prolongation of QTc interval from normal preoperative value to a maximum of 581 ms supports the diagnosis of acquired long QT syndrome.

aLQTS refers to prolongation of the QT interval induced by secondary factors such as medications, electrolyte disturbances, or bradycardia, which can provoke TdP (2). Female sex is a clear risk factor for aLQTS, and this sex difference is related to the fact that androgens shorten the QT interval while estrogens may prolong the QT interval and reduce repolarization reserve (3, 4). The patient in this case was female, had a normal preoperative electrocardiogram, and developed intraoperative bradycardia, hypokalemia (3.4 mmol/L), and hypocalcemia (ionized calcium concentration 0.98 mmol/L), with postoperative progressive prolongation of the QTc interval (Figure 2). Studies have confirmed that cardiac arrest itself can independently cause QTc prolongation (5), but according to the 2022 ESC Guidelines for the management of patients with ventricular arrhythmias, a QTc interval ≥460 ms is sufficient for the diagnosis of LQTS in patients who have experienced cardiac arrest (6). The patient in this case experienced intraoperative cardiac arrest and had a significantly prolonged postoperative QTc interval, meeting the electrocardiographic diagnostic criteria. At the same time, there were clear reversible precipitating factors including medications, electrolyte disturbances, and bradycardia, and no family history of congenital LQTS or related medical history. Therefore, the final diagnosis was aLQTS.

### 3.2 Electrolyte disturbances and perioperative aLQTS

Electrolyte disturbances are closely associated with aLQTS. Hypokalemia is one of the most common precipitating factors for aLQTS (7). In this patient, the intraoperative serum potassium dropped to 3.4 mmol/L, which was mildly below the lower limit of normal. This might be related to the administration of sodium bicarbonate immediately after cardiac arrest, which may have lowered the serum potassium level. Hypocalcemia (ionized calcium concentration 0.98 mmol/L) prolongs the action potential plateau phase and further synergizes with hypokalemia to exacerbate QT prolongation (8). Studies have confirmed that a decrease in serum magnesium level is significantly associated with QTc interval prolongation, but it is not an independent influencing factor (9). In this patient, electrolyte measurements were obtained after resuscitation from cardiac arrest. It cannot be excluded that electrolyte abnormalities may have already existed before, or may be related to perioperative hyperventilation, catecholamine surge, and redistribution during cardiac arrest/resuscitation. Unfortunately, the serum magnesium concentration was not measured during this event.

### 3.3 Bradycardia-dependent TdP

Bradycardia is an important trigger for torsade de pointes in patients with acquired long QT syndrome (10). Bradycardia can prolong the action potential duration of cardiomyocytes, induce bradycardia-dependent early afterdepolarizations and triggered activity, further significantly prolong the QT interval after long pauses, and increase repolarization dispersion, thereby forming the electrophysiological basis for TdP initiation and re-entry (11, 12). Dexmedetomidine is a highly selective α2-adrenoceptor agonist widely used for perioperative sedation. Studies have shown that at clinical doses, dexmedetomidine can mildly prolong the QT interval and should be used cautiously in the presence of other risk factors for arrhythmias (13). Another clinical study has confirmed that when the heart rate drops to ≤60 beats per minute, the QT interval can be significantly prolonged and the risk of TdP is markedly increased (14). In this patient, after administration of a dexmedetomidine loading dose of 80 μg (approximately 1 μg/kg), the heart rate was maintained at 52~57 beats per minute, which was already below the above-mentioned critical heart rate, and the QT interval was prolonged. In the setting of reduced repolarization reserve, dexmedetomidine-related bradycardia may have contributed to the occurrence of arrhythmia. Therefore, against the background of reduced repolarization reserve and a heart rate already below 60 beats per minute, dexmedetomidine-related bradycardia was very likely a key factor in triggering the arrhythmia in this patient.

### 3.4 Prevention strategies for survivors of cardiac arrest due to aLQTS

Survivors of cardiac arrest due to aLQTS should receive secondary prevention measures, the core of which is to reduce the risk of recurrent malignant ventricular arrhythmias and the incidence of sudden death. Implantation of an implantable cardioverter-defibrillator is recommended. In addition, lifestyle guidance should be provided simultaneously, avoiding triggers including the use of QT-prolonging medications and maintaining electrolyte balance, and beta-blockers should be incorporated as basic therapy (2, 6). Studies have shown that mexiletine can significantly shorten the QTc interval and suppress refractory TdP, and mexiletine may be added for TdP that recurs repeatedly despite beta-blocker therapy (15). For patients who do not respond adequately to the above treatment measures, left cardiac sympathetic denervation has a clear effect in reducing the risk of cardiac events and can be used as an adjunctive therapy (2, 16). Although the postoperative QTc interval returned to normal in this patient, long-term follow-up and careful consideration of the above prevention strategies are still required.

## 4 Conclusion

aLQTS is one of the important causes of perioperative cardiac arrest. Female sex, electrolyte disturbances, bradycardia, and QT-prolonging medications can act synergistically as triggers. Comprehensive interventions including early recognition of abnormal electrocardiographic signals, timely resuscitation, and correction of precipitating factors are key to improving prognosis. Anesthesiologists and nursing staff should enhance their awareness of aLQTS, thereby reducing the risk of perioperative cardiac arrest.

## Data Availability

The original contributions presented in the study are included in the article/supplementary material, further inquiries can be directed to the corresponding authors.
