# Correction: Cardiac arrest due to acquired long QT syndrome during gynecologic laparoscopy: a case report

**DOI:** 10.3389/fmed.2026.1954043

**Published:** 2026-08-12

**Authors:** Wenwen Wang, Ruijing Wang, Guangwei Li, Yun Zhang

**Affiliations:** 1 Department of Anesthesiology, The 960th Hospital of PLA, Jinan, Shandong, China; 2 Department of Vascular Surgery, The 960th Hospital of PLA, Jinan, Shandong, China; 3 Department of Anesthesiology, Jinan Municipal Hospital of Traditional Chinese Medicine, Jinan, Shandong, China

**Keywords:** cardiac arrest, dexmedetomidine, female, hypokalemia, long QT syndrome, perioperative period

Affiliation Department of Anesthesiology, The 960th Hospital of PLA, Jinan, Shandong, China was omitted from the paper. This affiliation has now been added for author Wenwen Wang.

Affiliation Department of Vascular Surgery, The 960th Hospital of PLA, Jinan, Shandong, China was erroneously included in the paper. This affiliation has now been removed for author Wenwen Wang.

The original version of this article has been updated.

